# Calculation of Overall Hospital Quality Star Ratings With and Without Inclusion of the Peer Grouping Step

**DOI:** 10.1001/jamanetworkopen.2024.11933

**Published:** 2024-05-16

**Authors:** Cameron J. Gettel, Kyle Bagshaw, Li Qin, Zhenqiu Lin, Eve Rothenberg, Prince Omotosho, Demetri P. Goutos, Jeph Herrin, Lisa G. Suter, Michelle Schreiber, Lee A. Fleisher, Raquel Myers, Steven B. Spivack, Arjun K. Venkatesh

**Affiliations:** 1 Department of Emergency Medicine, Yale School of Medicine, New Haven, Connecticut; 2 Center for Outcomes Research and Evaluation, Yale New Haven Health Services Corporation, New Haven, Connecticut; 3 Section of Cardiology, Department of Medicine, Yale School of Medicine, New Haven, Connecticut; 4 Department of Health Law, Policy, and Management, Boston University School of Public Health, Boston, Massachusetts; 5 Medicine, Yale School of Medicine, New Haven, Connecticut; 6 US Centers for Medicare & Medicaid Services, Department of Health and Human Services, Baltimore, Maryland; 7 The Lewin Group, Falls Church, Virginia

## Abstract

**Importance:**

The Centers for Medicare & Medicaid Services (CMS) Overall Star Rating is widely used by patients and consumers, and there is continued stakeholder curiosity surrounding the inclusion of a peer grouping step, implemented to the 2021 Overall Star Rating methods.

**Objective:**

To calculate hospital star rating scores with and without the peer grouping step, with the former approach stratifying hospitals into 3-, 4-, and 5-measure group peer groups based on the number of measure groups with at least 3 reported measures.

**Design, Setting, and Participants:**

This cross-sectional study used Care Compare website data from January 2023 for 3076 hospitals that received a star rating in 2023. Data were analyzed from April 2023 to December 2023.

**Exposure:**

Peer grouping vs no peer grouping.

**Main Outcomes and Measures:**

The primary outcome was the distribution of star ratings, with 1 star being the lowest-performing hospitals and 5 stars, the highest. Analyses additionally identified the number of hospitals with a higher, lower, or identical star rating with the use of the peer grouping step compared with its nonuse, stratified by certain hospital characteristics.

**Results:**

Among 3076 hospitals that received a star rating in 2023, most were nonspecialty (1994 hospitals [64.8%]), nonteaching (1807 hospitals [58.7%]), non–safety net (2326 hospitals [75.6%]), non–critical access (2826 hospitals [91.9%]) hospitals with fewer than 200 beds (1822 hospitals [59.2%]) and located in an urban geographic designations (1935 hospitals [62.9%]). The presence of the peer grouping step resulted in 585 hospitals (19.0%) being assigned a different star rating than if the peer grouping step was absent, including considerably more hospitals receiving a higher star rating (517 hospitals) rather than a lower (68 hospitals) star rating. Hospital characteristics associated with a higher star rating included urbanicity (351 hospitals [67.9%]), non–safety net status (414 hospitals [80.1%]), and fewer than 200 beds (287 hospitals [55.6%]). Collectively, the presence of the peer grouping step supports a like-to-like comparison among hospitals and supports the ability of patients to assess overall hospital quality.

**Conclusions and Relevance:**

In this cross-sectional study, inclusion of the peer grouping in the CMS star rating method resulted in modest changes in hospital star ratings compared with application of the method without peer grouping. Given improvement in face validity and the close association between the current peer grouping approach and stakeholder needs for peer-comparison, the current CMS Overall Star Rating method allows for durable comparisons in hospital performance.

## Key Points

**Question:**

What are the implications of applying a peer grouping step on hospitals’ Overall Star Ratings?

**Finding:**

In this cross-sectional study of 3076 hospitals that received a star rating in 2023, presence of the peer grouping step resulted in 585 hospitals (19.0%) being assigned a different star rating than if the peer grouping step was absent, including considerably more hospitals having a higher star rating (517 hospitals) than a lower star rating (68 hospitals).

**Meaning:**

These findings suggest that the inclusion of a star ratings peer grouping allows an updated comparison of quality between hospitals and better supports the ability of patients to assess overall hospital quality.

## Teaser

This cross-sectional study examines Centers for Medicare & Medicaid Services (CMS) Overall Star Rating scores with vs without the peer grouping step adopted by CMS in 2021 to ensure that star ratings reflect comparisons between hospitals with similar patterns of reported quality measures.

## Introduction

Care Compare is the health care comparison website created by the Centers for Medicare & Medicaid Services (CMS) to offer patients and caregivers information on quality of health care systems and practitioners.^1^ CMS introduced the Overall Hospital Quality Star Rating (hereafter, *Overall Star Rating*) in 2016 as a summary score of the quality measures reported on Care Compare. The Overall Star Rating assigns hospitals 1 to 5 stars based on their overall performance, which is intended to be easily interpreted by patients and consumers.^2^ The visibility of the Overall Star Rating has been shown to contribute to patient decision-making and has garnered high levels of attention from hospitals seeking to improve scores to attract patients.^3,4^

Some critics of the rating system have argued that earlier versions of the Overall Star Rating were biased toward certain key hospital characteristics, including patient volume and hospital size, academic teaching status, specialty, critical access status, and the proportion of patients with low income seen at the hospital.^5,6,7,8,9,10,11^ They noted that certain hospitals may have benefited by being scored on only measures related to procedures commonly performed or conditions most often treated, while not being scored on measures for other conditions or procedures the hospital was less experienced in due to low patient volumes. With all hospitals scored together in prior versions of the Overall Star Rating, these hospital measure exclusions may have advantaged or disadvantaged certain hospitals based on heterogeneous hospital characteristics, such as patient mix and service lines. These differences raised the possibility that some hospitals would be classified with a different star rating had they been compared only with hospitals scored on a similar type and number of measures.^12^ A proposed solution was peer grouping, in which hospitals receiving star ratings would be compared with peers with similar patterns of reported quality measures.

In January 2021, CMS published version 4.1 of the Overall Star Ratings method, with several significant methodological updates intended to address prior concerns and improve comparability between hospitals.^13^ Included in these changes was the incorporation of a peer grouping step, acknowledging the fact that hospitals may be scored on different types and numbers of measures based on their size, patient volume, services provided, and patient mix. This update was meant to allow fair comparison among hospitals included in CMS programs that reflected the overall quality of like hospitals. To our knowledge, there are no published data on how hospital star ratings are influenced with the presence of a peer grouping step. Therefore, we sought to assess how the incorporation of the peer grouping step changed 2023 hospital star ratings.

## Methods

This cross-sectional was approved by the Yale University institutional review board. This study was deemed exempt from informed consent as it was covered by the Common Rule exemption described at 45 CFR 46.104(d)(4)(i). We followed the Strengthening the Reporting of Observational Studies in Epidemiology (STROBE) reporting guideline.

### Data Sources

We used the January 2023 Care Compare results as the primary dataset for analyses.^14^ Updated on a quarterly basis, Care Compare is a publicly available website that reports numerous process, structural, and outcome measures for acute inpatient and outpatient hospitals.^1^ Using the hospital-specific CMS Certification Number, we linked the Care Compare dataset to several additional sources to incorporate data regarding hospital characteristics. Specifically, we used the 2018 Hospital Provider Cost Report to obtain hospital specialty status, bed size, urban or rural designation, and disproportionate share percentage.^15^ We used the FY 2022 IPPS Final Rule Impact file to obtain hospital teaching status.^16^

### Overall Star Ratings Peer Grouping

To directly assess the peer grouping step, we calculated star rating assignments in 2 fashions, including the use and nonuse of peer grouping. Our calculation of star ratings was in accordance with published methods reports and otherwise identical.^13^ In brief, we included all 46 underlying measures within the 5 measure groups that comprise Overall Star Ratings: mortality (7 measures), readmission (11 measures), safety of care (8 measures), patient experience (8 measures), and timely and effective care (12 measures) (eMethods in Supplement 1). We calculated a measure group score based on the simple mean of standardized measure scores within the group for each hospital, followed by the determination of a hospital summary score as the weighted mean of each hospital’s measure group scores. For example, if a hospital had Care Compare scores for 8 measures within the safety of care measure group, then each of the standardized measure scores would contribute 12.5% toward the safety of care measure group score. Similarly, for a hospital with standardized measure scores for all 5 measure groups, then the safety of care measure group would contribute 22% toward the hospital summary score, in addition to the mortality, readmission, and patient experience measure groups contributing 22% each and the timely and effective care measure group contributing 12%.

We then applied CMS’s public reporting thresholds, requiring hospitals to have at least 3 measures in each of at least 3 measure groups (1 of which must be the mortality or safety of care group) to receive a star rating. For the approach with use of the peer grouping step, remaining hospitals were assigned to a peer group based on the number of measure groups in which the hospital had 3 or more reported measures, resulting in a 3-measure group peer group (peer group 3), a 4-measure group peer group (peer group 4), and a 5-measure group peer group (peer group 5). For the approach without the peer grouping step, all hospitals meeting the threshold were compared in a single group, reflecting the only difference between approaches. Independently within each peer group (when performed) or across all hospitals when peer grouping was not performed, we then used a k-means clustering algorithm to relatively classify hospitals into 5-star rating categories based on the overall summary score, with 1 star being the lowest-performing hospitals and 5 stars, the highest.

### Statistical Analysis

To better understand the composition of hospitals within each individual peer group, we described characteristics for each peer group of hospitals, including hospital specialty status, teaching status, safety-net status, critical access status, bed size, geography, and disproportionate share hospital patient percentage (calculated as [Medicare Supplemental Security Income days / total Medicare days] + [Medicaid, non-Medicare days / total patient days]). We identified teaching status as nonteaching if no residents were present, minor teaching if fewer than 100 residents were present, and major teaching if at least 100 residents were present. We identified hospitals as safety net status if they were publicly funded or if the hospital’s number of inpatient Medicaid discharges were 1 SD above the state mean.

We then identified the distribution of star ratings whether the peer grouping step was used or not used. We also identified the number of hospitals with a higher, lower, or identical star rating with the use of the peer grouping step compared with its nonuse to understand how the star ratings of hospitals with certain characteristics were influenced by the peer grouping step.

We further sought to describe the measure group reporting patterns that served as drivers for changes in star ratings to identify which measure groups were not frequently reported by hospitals in peer groups 3 and 4. Finally, we calculated the mean score within each measure group, stratified by peer group and star rating.

*P* values were 2-sided, and statistical significance was set at *P* < .05. Analyses were conducted using SAS version 9.4 (SAS Institute). Data were analyzed from April 2023 to December 2023.

## Results

Of 4654 hospitals with quality measures reported on Care Compare in January 2023, 3076 were assigned a star rating. Of hospitals that were assigned a star rating, most were nonspecialty (1994 hospitals [64.8%]), nonteaching (1807 hospitals [58.7%]), non–safety net (2326 hospitals [75.6%]), non–critical access (2826 hospitals [91.9%]) hospitals with fewer than 200 beds (1822 hospitals [59.2%]) and in urban geographic designations (1935 hospitals [62.9%]). Without the peer grouping step incorporated, 267 hospitals (8.7%) would have received 1 star, 734 hospitals (23.9%) would have received 2 stars, 965 hospitals (31.4%) would have received 3 stars, 800 hospitals (26.0%) would have received 4 stars, and 310 hospitals (10.0%) would have received 5 stars (eTable 1 in Supplement 1). With peer grouping applied, 250 hospitals (8.1%) hospitals received 1 star, 668 hospitals (21.7%) received 2 stars, 872 hospitals (28.3%) received 3 stars, 803 hospitals (26.1%) received 4 stars, and 483 hospitals (15.7%) received 5 stars. Peer group 5 was the largest (2420 hospitals [78.7% of all hospitals with a star rating]), followed by peer group 4 (462 hospitals [15.0%]) and peer group 3 (194 hospitals [6.3%]). Peer group 5 hospitals tended to be non–safety net status, non–critical access, urban, and have a greater number of beds (Table 1).

**Table 1.** Overall Star Ratings and Hospital Characteristics Stratified by Peer Group, January 2023

Characteristic	Hospitals by peer group, No. (%)		
	3 (n = 194)	4 (n = 462)	5 (n = 2420)
Star rating			
1	22 (11.3)	25 (5.4)	203 (8.4)
2	40 (20.6)	84 (18.2)	544 (22.5)
3	73 (37.6)	128 (27.7)	671 (27.7)
4	37 (19.1)	130 (28.1)	636 (26.3)
5	22 (11.3)	95 (20.6)	366 (15.1)
Specialty			
Specialty	58 (29.9)	77 (16.7)	819 (33.8)
Nonspecialty	127 (65.5)	353 (76.4)	1514 (62.6)
NA	9 (4.6)	32 (6.9)	7 (3.6)
Teaching			
Nonteaching	145 (74.7)	376 (81.4)	1286 (53.1)
Minor teaching	42 (21.6)	55 (11.9)	799 (33.0)
Major teaching	1 (0.5)	7 (1.5)	251 (10.4)
NA	6 (3.1)	24 (5.2)	84 (3.5)
Safety net status			
Non–safety net	124 (63.9)	309 (66.9)	1893 (78.2)
Safety net	61 (31.4)	119 (25.8)	436 (18.0)
NA	9 (4.6)	34 (7.4)	91 (3.8)
Critical access			
Noncritical access	127 (65.5)	295 (63.9)	2404 (99.3)
Critical access	67 (34.5)	67 (36.1)	16 (0.7)
Bed size, No.			
1-99	137 (70.6)	379 (82.0)	509 (21.0)
100-199	23 (11.9)	34 (7.4)	740 (30.6)
200-299	15 (7.7)	12 (2.6)	442 (18.3)
300-399	4 (2.1)	3 (0.6)	278 (11.5)
≥400	6 (3.1)	2 (0.4)	364 (15.0)
NA	9 (4.6)	32 (6.9)	87 (3.6)
Geography			
Urban	71 (36.6)	160 (34.6)	1704 (70.4)
Rural	114 (58.8)	270 (58.4)	629 (26.0)
NA	9 (4.6)	32 (6.9)	87 (3.6)
Disproportionate share hospital patient %, quintile^a^			
1	29 (31.2)	48 (23.1)	404 (19.1)
2	21 (22.6)	28 (13.5)	434 (20.6)
3	21 (22.6)	85 (40.9)	378 (17.9)
4	6 (6.5)	13 (6.3)	462 (21.9)
5	16 (17.2)	34 (16.3)	432 (20.5)

Abbreviations: DSH, disproportionate share hospital; NA, not available.

^a^ DSH quintiles are defined among the 2411 hospitals with a star rating and an operating DSH adjustment. Quintile 1 includes hospitals with the lowest DSH percentage.

Application of peer grouping resulted in 585 hospitals (19.0%) being assigned a different star compared with no peer grouping; no hospital’s star rating differed by more than 1 star. In peer group 3, a total of 24 hospitals had a higher star rating and 20 hospitals had a lower star rating with the use of peer grouping than without its use. In peer group 4, 48 hospitals had a lower star rating while none had a higher star rating with the use of peer grouping than without its use. Conversely, all 493 hospitals in peer group 5 had a higher star rating while none had a lower star rating with the use of peer grouping than without its use (Table 2).

**Table 2.** Overall Star Rating Distribution With and Without the Peer Grouping Step for January 2023 Care Compare Reporting Hospitals

Measure	Star rating if peer grouping used, No. (%)						
	1	2	3	4	5	Total No.
**Among hospitals in peer group 3**							
Star rating if peer grouping not used						
1	22 (61.1)	14 (38.9)	0	0	0	36
2	0	26 (72.2)	10 (27.8)	0	0	36
3	0	0	53 (100)	0	0	53
4	0	0	10 (27.0)	27 (73.0)	0	37
5	0	0	0	10 (31.3)	22 (68.8)	32
Total, No.	22	40	73	37	22	194
**Among hospitals in peer group 4**							
Star rating if peer grouping not used						
1	24 (100)	0	0	0	0	24
2	1 (1.4)	69 (98.6)	0	0	0	70
3	0	15 (12.5)	105 (87.5)	0	0	120
4	0	0	23 (16.0)	121 (84.0)	0	144
5	0	0	0	9 (8.7)	95 (91.3)	104
Total, No.	25	84	128	130	95	462
**Among hospitals in peer group 5** ^a^							
Star rating if peer grouping not used						
1	203 (98.1)	4 (1.9)	0	0	0	207
2	0	540 (86.0)	88 (14.0)	0	0	628
3	0	0	583 (73.6)	209 (26.4)	0	792
4	0	0	0	427 (69.0)	192 (31.0)	619
5	0	0	0	0	174 (100)	174
Total, No.	203	544	671	636	366	2420

^a^ An example interpretation is that among the 2420 hospitals in peer group 5, 209 hospitals would have received a star rating of 3 if peer grouping were not used but would have received a star rating of 4 if peer grouping were used.

With peer grouping applied, we identified modest shifts in Overall Star Ratings, stratified by key hospital characteristics. Of 68 hospitals receiving a lower star rating with peer grouping, most were rural, non–safety net hospitals and hospitals with low bed volume (1-99 beds). Of 517 hospitals receiving a higher rating with peer grouping present, most were urban, non–safety net hospitals and hospitals with a higher bed volume (Table 3).

**Table 3.** Star Rating Changes Based on Hospital Characteristics With Use vs Nonuse of a Peer Grouping Step^a^

Star ratings change	No. (%)										Total, No.
	Beds, No.					Safety net status		Location		Not available	
	1-99	100-199	200-299	300-399	≥400	Non–safety net	Safety-net	Urban	Rural		
−1	55 (80.9)	6 (8.8)	2 (2.9)	2 (2.9)	1 (1.5)	48 (70.6)	18 (26.5)	25 (36.8)	41 (60.3)	2 (2.9)	68
0	841 (33.8)	633 (25.4)	374 (15.0)	229 (9.2)	309 (12.4)	1864 (74.8)	516 (20.7)	1559 (62.6)	827 (33.2)	105 (4.2)	2491
+1	129 (25.0)	158 (30.6)	93 (18.0)	54 (10.4)	62 (12.0)	414 (80.1)	82 (15.9)	351 (67.9)	145 (28.1)	21 (4.1)	517
Total, No.	1025	797	469	285	372	2326	616	1935	1013	128	3076

^a^ An example interpretation is that 351 (67.9%) of the 517 hospitals that had a higher star rating after peer grouping step implementation were identified to be in an urban geography.

By definition, all 2420 hospitals in peer group 5 reported at least 3 measures in each of the measure groups. Most hospitals (71.6%) in peer group 3 did not meet reporting requirements for the patient experience measure group but almost universally were scored on at least 3 measures within the readmission and timely and effective care measure groups (Table 4). Stratified by peer group, hospitals receiving a greater number of stars tended to receive higher mean measure group scores (eTable 2 in Supplement 1).

**Table 4.** Measure Group Reporting by Hospitals Within Peer Groups

Measure group	Hospitals, No. (%)			
	All hospitals receiving a star rating	Peer group		
		3 Measures	4 Measures	5 Measures
Mortality	3009 (97.8)	161 (83.0)	428 (92.6)	2420 (100)
Safety of care	2995 (97.4)	144 (74.2)	431 (93.3)	2420 (100)
Readmission	3075 (100)	193 (99.5)	462 (100)	2420 (100)
Timely and effective care	3075 (100)	193 (99.5)	462 (100)	2420 (100)
Patient experience	2923 (95.0)	55 (28.4)	448 (97.0)	2420 (100)

## Discussion

In this cross-sectional study, we describe the application of a peer grouping step and the associated changes in the assignment of CMS’s overall hospital star rating. As 19% of hospitals receiving a 2023 star rating would have received a different rating without peer grouping (by no more than 1 star), our results show a modest change on hospitals’ assigned star ratings from the peer grouping step that groups hospitals with similar characteristics together. Incorporation of peer grouping within the star rating method allows comparison of hospitals for which a similar amount of measure information is reported and therefore likely have similar patient volumes and patient mixes. Overall, the peer grouping step provides an improvement to the star ratings assignment method by enhancing the comparability of hospitals receiving a star rating.

Most importantly, the presence of the peer grouping step outlined in this study is responsive to prior stakeholder concerns with earlier versions of the star rating method.^5,6,7,8,9,10,11,12^ A prior “Rating the Raters” assessment of 4 hospital quality rating systems identified that an earlier version of the star rating method, without peer grouping present, resulted in the heterogeneous collection of hospitals into a single group.^12^ Resultantly, large academic medical centers that reported nearly all of the underlying measures within the star ratings method were compared with critical access hospitals reporting considerably fewer. As part of discussions with stakeholders regarding the comparability of hospital star ratings, peer grouping was discussed and gained face validity in technical expert panels, patient advocate workgroups, organizational leadership workgroups, and public comment periods. Stakeholders identified that larger hospitals with more diverse patient mix and service mix, such as large urban teaching hospitals, frequently report a greater number of measures, and should therefore be compared within a more directly comparable group.^17^

Stratification by hospital characteristics showed noticeable differences in the makeup of each of the 3 peer groups, particularly apparent for bed size, geography, and critical access status. This finding is aligned with and expands on the work by Chung et al^11^ that assessed the 2017 star ratings program which, prior to peer grouping, used a cluster analytic approach to outline a method that grouped hospitals “that take similar tests,” as they report similar measures and similar numbers of measures.^11^ The 3 clusters had patterns of unique characteristics, including teaching status, size, and patient mix index, yet the work was limited by potential complexity and difficulty for stakeholders to understand, as the number and composition of clusters could change every year. The current approach to peer grouping in the Overall Star Rating version 4.1 method captures the benefit of a considerable degree of proportional consistency between hospital characteristics and the number of measure groups with multiple measures, while also resulting in an easily interpretable 3 to 5 measure group peer group stratification.

Presence of the peer grouping step resulted in 3 noteworthy trends. First, a review of the overall outcome found that a considerably greater number of hospitals received a higher star rating (517 hospitals) than a lower rating (68 hospitals) when ratings were calculated using peer grouping. Second, all hospitals in peer group 4 with a star rating difference received a lower rating with the use of peer grouping, while all hospitals in peer group 5 with a star ratings difference received a higher rating. Based on hospital characteristics, the lower ratings were observed for hospitals that were predominantly small (low number of beds), rural, or teaching. Prior literature speculated that some hospitals with these characteristics may have had an advantage in earlier versions of Overall Star Ratings due to not being scored on measures with low patient volumes.^5^ Based on our findings, use of the peer grouping step may result in a more appropriate comparison among similar hospitals. Third, the more granular assessment of the measure group reporting frequencies can help answer the question of what is driving change in the star ratings with addition of the peer grouping step. In peer group 4, the mortality measure group was most commonly (7.4%) omitted from public reporting due to insufficient patient volumes; in peer group 3, the safety of care and patient experience measure groups are most commonly omitted (25.8% and 71.6%, respectively). This provides evidence of a greater level of homogeneity in the submitted measure groups when making comparisons among hospitals with the presence of the peer grouping step.

### Limitations

We recognize several limitations of our study. First, the Overall Star Rating only includes hospitals that meet the reporting requirements for a sufficient selection of the underlying measures reported on Care Compare. Thus, our findings do not account for hospitals that did not meet inclusion criteria to receive a star rating. However, because our focus was only on hospitals that were included in publicly reported Overall Star Ratings, this is a minor limitation. Second, we recognize some incongruity between measurement results (January 2023), and the date at which some hospital characteristics were defined (2018). It is possible that characteristics of included hospitals may have changed between 2018 and 2023. Third, analyses did not cross-validate to conclude whether the new method of Overall Star Rating is more or less accurate but rather assessed agreement between the use and nonuse of the peer grouping step. Fourth, in accordance with the overall hospital star ratings methods, hospitals were assigned to a peer group based on the number of measure groups in which the hospital had 3 or more reported measures. While this approach was determined through several years of stakeholder engagement discussions, other methodologic approaches may be considered to explicitly group peer hospitals together, such as hospital characteristics (eg, specialty status, teaching status, critical access status). However, recent CMS final rules have shown that the number of measure groups reported by a hospital is closely associated with certain characteristics and is therefore a valid approach^18^; as an example, urban teaching hospitals with a greater number of beds are more frequently within peer group 5 compared with rural nonteaching hospitals with a smaller number of beds.

## Conclusions

In this cross-sectional study, the inclusion of a peer grouping step in the overall hospital star ratings method allowed an updated comparison of quality between hospitals. Separating hospitals based on the number of measure groups reported ensures that hospitals are more often compared against similar hospitals and supports the ability of patients to identify overall hospital quality.

## Notes

Supplement 1. **eMethods.** Measures Included in the 2023 Overall Star Ratings by Measure Group **eTable 1.** Star Rating Overall Distribution With and Without the Peer Grouping Step for January 2023 Care Compare Reporting Hospitals **eTable 2.** Average Measure Group Scores Stratified by Peer Group and Star Rating

Supplement 2.
Data Sharing Statement
