# Author Omitted

**DOI:** 10.1001/jamanetworkopen.2024.26551

**Published:** 2024-07-15

In the Original Investigation titled “Calculation of Overall Hospital Quality Star Ratings With and Without Inclusion of the Peer Grouping Step,”^1^ published May 16, 2024, Steven B. Spivack, PhD, was omitted among the coauthors owing to an oversight from the corresponding author. This article has been corrected.^1^
