# Supplementary material for: Calculation of Overall Hospital Quality Star Ratings With and Without Inclusion of the Peer Grouping Step
Source: JAMA Netw Open. 2024 May 16;7(5):e2411933. doi: 10.1001/jamanetworkopen.2024.11933 (PMC11099678; doi:10.1001/jamanetworkopen.2024.11933)
Supplement: Supplement 1. — eMethods. Measures Included in the 2023 Overall Star Ratings by Measure Group eTable 1. Star Rating Overall Distribution With and Without the Peer Grouping Step for January 2023 Care Compare Reporting Hospitals eTable 2. Average Measure Group Scores Stratified by Peer Group and Star Rating [file jamanetwopen-e2411933-s001.pdf]

Supplemental Online Content

Gettel CJ, Bagshaw K, Qin L, et al. Calculation of overall hospital quality star ratings with and without inclusion of the peer grouping step. *JAMA Netw Open*. 2024;7(5):e2411933. doi:10.1001/jamanetworkopen.2024.11933

eMethods. Measures Included in the 2023 Overall Star Ratings by Measure Group

eTable 1. Star Rating Overall Distribution With and Without the Peer Grouping Step for January 2023 Care Compare Reporting Hospitals

eTable 2. Average Measure Group Scores Stratified by Peer Group and Star Rating

This supplemental material has been provided by the authors to give readers additional information about their work.

© 2024 Gettel CJ et al. *JAMA Network Open.*

eMethods. Measures included in the 2023 Overall Star Ratings (N=46) by measure group

Mortality

1. MORT-30-AMI: 30-day death rate for heart attack patients
2. MORT-30-CABG: Death rate for coronary artery bypass graft surgery patients
3. MORT-30-COPD: Death rate for chronic obstructive pulmonary disease (COPD) patients
4. MORT-30-HF: 30-day death rate for heart failure patients
5. MORT-30-PN: 30-day death rate for pneumonia patients
6. MORT-30-STK: Death rate for stroke patients
7. PSI 04: Death rate among surgical inpatients with serious treatable complications

Safety of Care

1. COMP-HIP-KNEE: Rate of complications for hip and knee replacement patients
2. HAI-1: Central-line associated bloodstream infection (CLABSI)
3. HAI-2: Catheter-associated urinary tract infection (CAUTI)
4. HAI-3: Surgical site infection from colon surgery (SSI: Colon)
5. HAI-4: Surgical site infection from abdominal hysterectomy (SSI-abdominal hysterectomy)
6. HAI-5: Methicillin-resistant staphylococcus aureus (or MRSA) blood infections (Antibiotic-resistant blood infections)
7. HAI-6: Clostridium difficile (or C.diff.) infections (Intestinal infections)
8. PSI 90: Patient Safety and Adverse Events Composite

Readmission

1. EDAC-30-AMI: Acute myocardial infarction excess days in acute care (EDAC)
2. EDAC-30-HF: Heart failure excess days in acute care (EDAC)
3. EDAC-30-PN: Pneumonia excess days in acute care (EDAC)
4. READM-30-CABG: Rate of unplanned readmission after coronary artery bypass graft (CABG) surgery
5. READM-30-COPD: Rate of unplanned readmission for chronic obstructive pulmonary disease patient
6. READM-30-Hip-Knee: 30-day rate of readmission for hip and knee replacement patients
7. READM-30-HOSP-WIDE: Rate of readmission after discharge from hospital
8. OP-32: Facility 7-day risk standardized hospital visit rate after outpatient colonoscopy
9. OP-35 ADM: Admissions visits for patients receiving outpatient chemotherapy
10. OP-35 ED: Emergency department (ED) visits for patients receiving outpatient chemotherapy
11. OP-36: Hospital visits after hospital outpatient surgery

Patient Experience

1. H-COMP-1: Communication with nurses
2. H-COMP-2: Communication with doctors
3. H-COMP-3: Responsiveness of hospital staff
4. H-COMP-5: Communication about medicines
5. H-COMP-6: Discharge information
6. H-COMP-7: Care transition
7. H-HSP-RATING Hospital rating (Q21) + H-RECMND: Willingness to recommend hospital (Q22) / 2
8. H-CLEAN-HSP Cleanliness of hospital environment (Q8) + H-QUIET-HSP Quietness of hospital environment (Q9) / 2

Timely and Effective Care

1. IMM-3: Percent of healthcare workers vaccinated against Influenza
2. OP-22: Percentage of patients who left the emergency department before being seen
3. OP-23: Percentage of patients who came to the emergency department with stroke symptoms who received brain scan results within 45 minutes of arrival
4. OP-29: Appropriate follow-up interval for normal colonoscopy in average risk patients

© 2024 Gettel CJ et al. *JAMA Network Open.*

5. PC-01: Percent of newborns whose deliveries were scheduled too early (1-3 weeks early), when a scheduled delivery was not medically necessary
6. SEP-1: Percentage of patients who received appropriate care for severe sepsis and septic shock
7. OP-3b: Average number of minutes before outpatients with chest pain or possible heart attack who needed specialized care were transferred to another hospital
8. OP-18b: Average time patients spent in the emergency department before being sent home
9. OP-8: Outpatients with low back pain who had an MRI without trying recommended treatments first, such as physical therapy
10. OP-10: Outpatient CT scans of the abdomen that were “combination” (double) scans
11. OP-13: Medicare patients who got cardiac imaging stress tests to screen for surgical risk before low-risk outpatient surgery
12. HCP COVID-19: COVID-19 vaccination coverage among HCPs

© 2024 Gettel CJ et al. *JAMA Network Open.*

eTable 1. Star rating overall distribution with and without the peer grouping step for January 2023 Care Compare reporting hospitals

		Star Rating if Peer Grouping Used					
		1	2	3	4	5	Total
Star Rating if Peer Grouping Not Used	1	249 (93.3)	18 (6.7)	0	0	0	267
	2	1 (0.1)	635 (86.5)	98 (13.4)	0	0	734
	3	0	15 (1.6)	741 (76.8)	209 (21.7)	0	965
	4	0	0	33 (4.1)	575 (71.9)	192 (24.0)	800
	5	0	0	0	19 (6.1)	291 (93.9)	310
	Total	250	668	872	803	483	3076

© 2024 Gettel CJ et al. *JAMA Network Open.*

eTable 2. Average measure group scores stratified by peer group and star rating

Measure Group	Peer Group	Star Rating[a]				
		1	2	3	4	5
**Mortality**	3	-0.97	-0.70	-0.29	0.16	0.79
	4	-1.05	-0.43	-0.30	0.16	0.61
	5	-0.59	-0.32	0.01	0.43	0.93
**Safety of Care**	3	-1.74	-0.24	0.14	0.62	0.80
	4	-2.31	-0.78	-0.10	0.29	0.87
	5	-0.70	-0.27	-0.03	0.20	0.47
**Readmission**	3	-1.45	-0.65	0	0.59	1.36
	4	-1.07	-0.35	0.02	0.29	0.96
	5	-1.38	-0.61	-0.14	0.20	0.56
**Timely and Effective Care**	3	-0.64	-0.80	0.02	0.44	0.35
	4	-0.53	-0.17	0.07	0.11	0.41
	5	-0.67	-0.35	-0.15	-0.01	0.14
**Patient Experience**	3	-1.86	-0.58	-0.13	0.43	0.78
	4	-0.02	-0.12	0.40	0.92	1.24
	5	-1.32	-0.73	-0.32	0.07	0.58

Notes: [a]For each hospital, standardized individual measure scores are averaged to obtain a measure group score, which is subsequently standardized again to allow for comparisons across hospitals. An example interpretation is that among hospitals in Peer Group 5, the average Mortality measure group scores ranged from -0.59 (1 star hospitals) to 0.93 (5 star hospitals).

© 2024 Gettel CJ et al. *JAMA Network Open.*
