# Supplementary material for: Calculation of Overall Hospital Quality Star Ratings With and Without Inclusion of the Peer Grouping Step
Source: JAMA Netw Open. 2024 May 16;7(5):e2411933. doi: 10.1001/jamanetworkopen.2024.11933 (PMC11099678; doi:10.1001/jamanetworkopen.2024.11933)
Supplement: Supplement 2. — Data Sharing Statement [file jamanetwopen-e2411933-s002.pdf]

Data Sharing Statement

Gettel. Calculation of Overall Hospital Quality Star Ratings With and Without Inclusion of the Peer Grouping Step. *JAMA Netw Open*. Published May 16, 2024.
doi:10.1001/jamanetworkopen.2024.11933

Data
Data available: Yes
Data types: Data (not involving human participants), Data dictionary
How to access data: Email - cmsstarratings@yale.edu
When available: With publication

Supporting Documents
Document types: None

Additional Information
Who can access the data: Researchers whose proposed use of the data has been reviewed and approved by the Star Ratings team.
Types of analyses: For specific purposes upon approval by the Star Ratings team.
Mechanisms of data availability: After approval of proposal.
